# Malignant Middle Cerebral Artery Infarct Caused by Eagle’s Syndrome

**DOI:** 10.7759/cureus.47205

**Published:** 2023-10-17

**Authors:** Mohammad Umair Sarwar, Muhammad Furrukh, Mohammad Ali Tabrez, Aqil Kannar, Muhammad Ali Sumbal, Muhammad Haseeb

**Affiliations:** 1 Medicine, Dorset County Hospital, Dorchester, GBR; 2 Medicine, Holy Family Hospital, Rawalpindi, PAK; 3 Radiology, Dorset County Hospital, Dorchester, GBR; 4 General Medicine, Dorset County Hospital, Dorchester, GBR; 5 Medicine, Balfour Hospital, Kirkwall, GBR; 6 Dentistry, Sharif Medical & Dental College, Lahore, PAK

**Keywords:** acute ischemic infarct, ischemic cerebrovascular disease, internal carotid artery occlusion, internal carotid artery (ica), carotid arterial dissection, non traumatic carotid artery dissection, elongated styloid syndrome, eagle's syndrome

## Abstract

Eagle’s syndrome is characterised by elongation of the styloid process. The elongated styloid process can cause symptoms like dysphagia, facial or neck pain, syncope, visual changes, etc. In severe cases, it may cause a rupture or dissection of the carotid artery, which can lead to intracranial thrombo-embolism and ischemic stroke. We report a case of a 57-year-old male presenting with dysarthria and mild left-sided body weakness. An initial non-contrast computed tomography (CT) scan showed a possible right internal carotid artery thrombus. He developed worsening left-sided weakness and gaze palsy one day after the admission. Repeated CT brain and intracranial angiography were arranged, which showed significant oedema with mass effect and right internal carotid artery dissection with thrombus. He underwent decompressive craniectomy. An enlarged styloid process measuring 4.53 cm in close proximity to the cervical vasculature was also noted. He was not deemed an appropriate candidate for styloidectomy. Due to residual left-sided weakness, he had to take early retirement. He underwent extensive rehabilitation and was able to mobilize with the help of a quad stick after a period of nine months. At the five-year follow-up, there were no characteristic symptoms of Eagle's syndrome and he was mobilizing without support.

## Introduction

Eagle’s syndrome is characterised by the elongation of the styloid process or calcification of the stylomandibular or stylohyoid ligaments [1]. The length of the styloid process varies greatly and is considered normal up to 3 cm in adults. The tip of the styloid process is located between the external and internal carotid arteries, slightly lateral to the tonsillar fossa. The elongated styloid process can cause irritation or impingement on the adjacent neurovascular structures, producing a vast array of symptoms. Symptoms can range from dysphagia, facial, neck or throat pain to syncope, visual symptoms, and cerebrovascular accidents [2]. The usual mechanism of cerebrovascular accidents is the irritation from the elongated styloid process, which causes rupture or dissection of the carotid artery, leading to intracranial thrombo-embolism and ischemic stroke [3]. Here, we present a case of malignant middle cerebral artery infarction secondary to carotid artery dissection (CAD) caused by a unilaterally elongated styloid process.

## Case presentation

**Figure 1:**
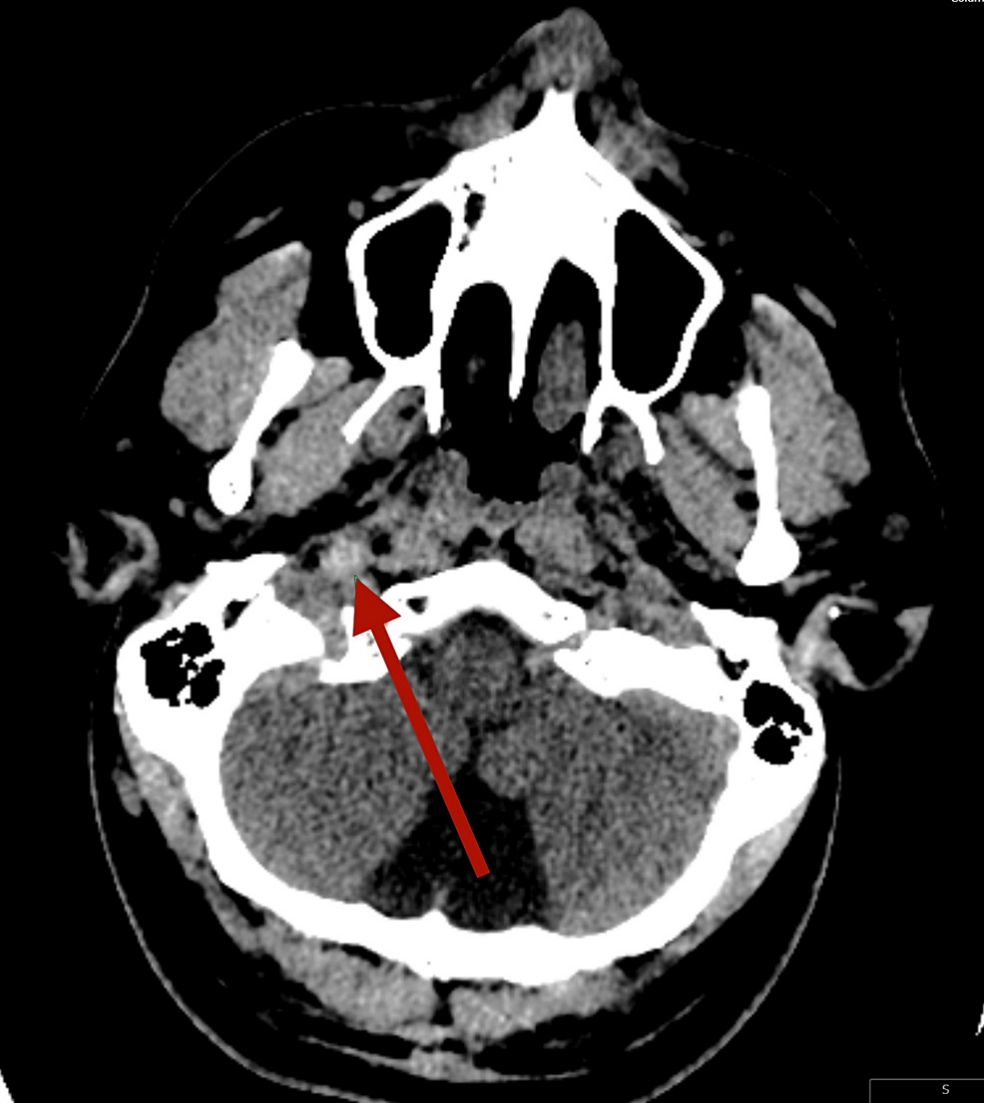
Non-contrast CT scan showing hyperdensity in right ICA at skull base ICA: internal carotid artery

**Figure 2:**
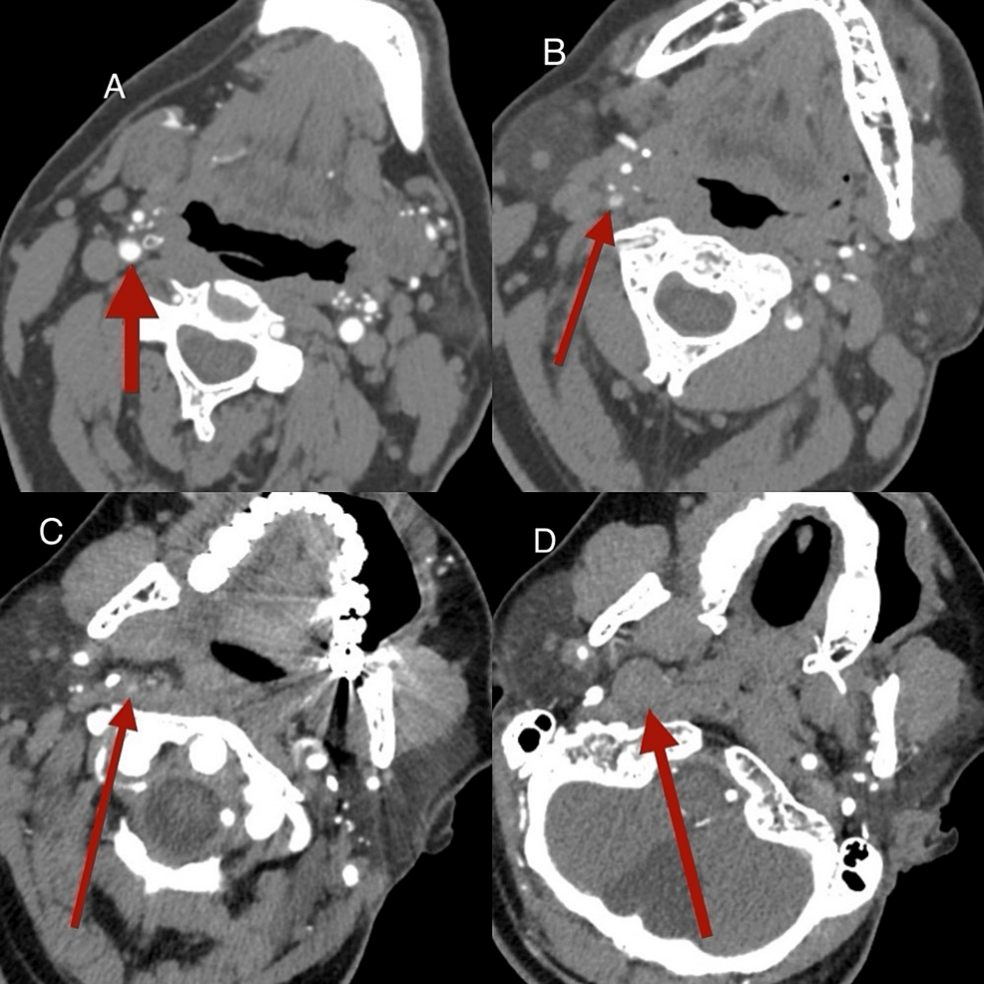
CT showing ICA dissection and thrombus (A) Normal carotid artery below the level of dissection; (B) Beginning of right ICA dissection: attenuation of the contrast product in the proximal segment and reduction of arterial lumen; (C) Right ICA dissection just medial to the large styloid process; (D) Enlarged and completely thrombosed right ICA at the skull base, characterized by absence of contrast product ICA: internal carotid artery

A 57-year-old male with no significant comorbidities presented to the emergency department with dysarthria and left-sided body weakness. He woke up with these symptoms and there was no preceding history of rapid head movements, fall, or trauma. On examination, vital signs were within normal limits. Neurological examination revealed reduced power in the left upper and lower limbs with decreased sensation. National Institute of Health Stroke Scale (NIHSS) on arrival was 8. Initial non-contrast computed tomography (CT) of the head did not show any haemorrhage or infarct but it did show high attenuation material within the right internal carotid artery (ICA) at the skull base, which was possibly extending into the region of the cavernous segment of the ICA, likely representing fresh thrombus (Figure 1). Magnetic resonance imaging could not be arranged due to retained pacemaker lead material. Unfortunately, he was out of the time window for thrombolysis and thrombectomy. He was commenced on aspirin 300 mg once a day. The next day, he developed dense left-sided weakness and right gaze palsy. His NIHSS increased to 20 from 8. Repeat CT head with aortic arch and carotid angiography revealed occlusion of right ICA from carotid bifurcation to the skull base (Figure 2). There was also opacification of the M1 segment of the middle cerebral artery with occlusion of distal branches.

**Figure 3:**
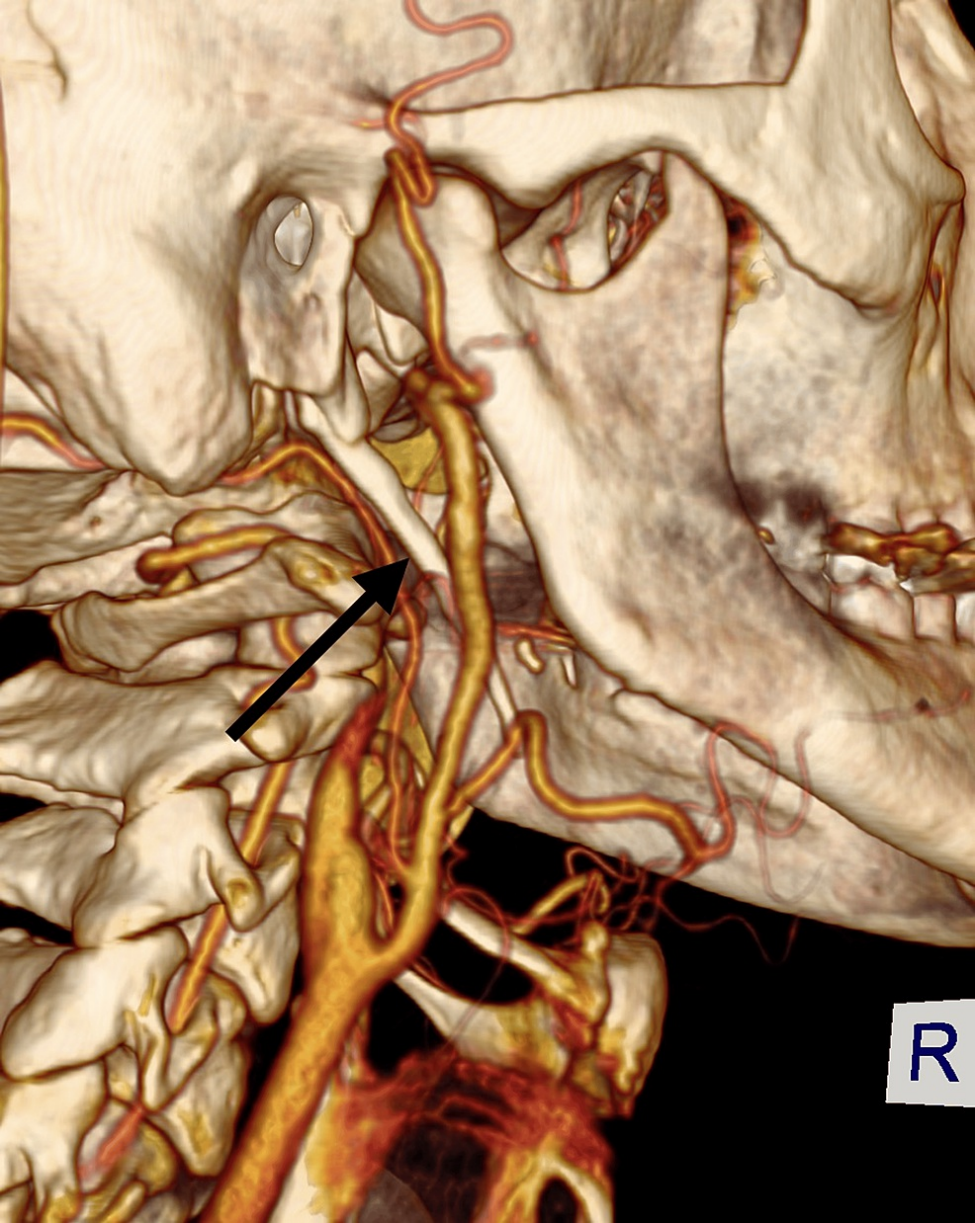
Three-dimensional CT reconstruction: black arrow pointing towards elongated styloid process

**Figure 4:**
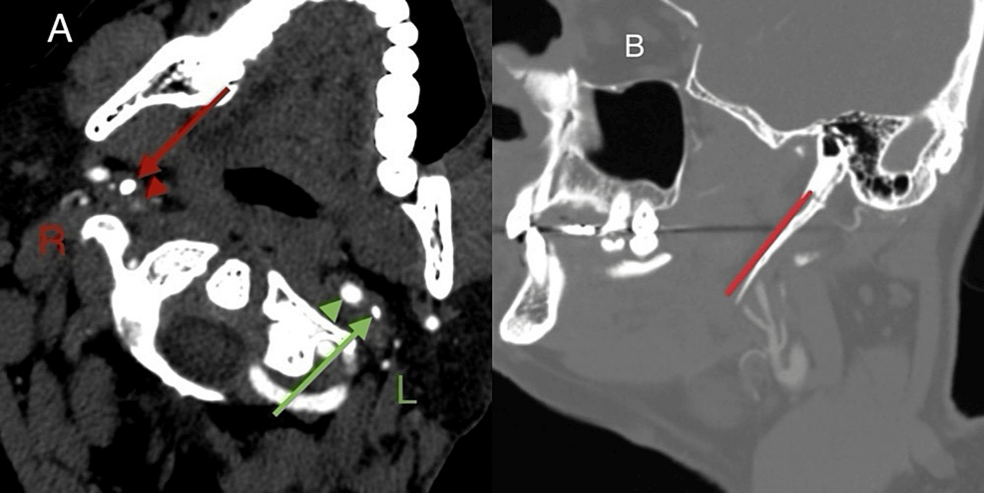
CT showing elongated styloid process in close proximity to right ICA (A) On right side, red arrow is showing thick styloid process adjacent to thrombosed ICA (red arrow head),  as compared to the left side where green arrow is showing thin styloid process adjacent to normal ICA (green arrow head); (B) Red line showing elongated styloid process (length=4.53cm). ICA: internal carotid artery

The patient was transferred to the neurosurgical regional centre for further management where he underwent decompressive craniectomy due to significant oedema, causing mass effect. After craniectomy, he was shifted to the stroke rehabilitation unit, where he received extensive input from occupational therapists and physiotherapists. Images were reviewed in the radiology multidisciplinary meeting and an elongated styloid process on the right was identified with a length of 4.53 cm (Figures 3, 4). In the absence of any atherosclerotic changes and other systemic illnesses, an elongated styloid process was considered to be the likely cause of the carotid dissection. The case was discussed with an otorhinolaryngologist but the patient was not deemed appropriate for any surgical intervention. He showed gradual improvement in functional status and was discharged home with follow-up from the community physiotherapy team. Due to significant residual weakness, he had to take early retirement. He was able to mobilize with the help of a quad stick after a period of nine months. At the five-year follow-up, he could mobilise without support and was able to carry out activities of daily living independently.

## Discussion

Stroke is a major cause of morbidity and mortality worldwide. It accounts for 5.2% of all fatalities globally. Ischemic stroke is the most common type of stroke and results from an obstruction to the arterial circulation [4]. Approximately 50% of ischemic strokes are caused by arteriosclerosis, while 25% are produced by lacunar infarcts, 20% have cardiogenic causes, and the remaining 5% result from other unusual factors including vasculitis and arterial dissection [5,6]. Although uncommon, CAD is a recognized cause of ischemic stroke. Connective tissue problems, acquired illnesses including infection, hypertension, and structural abnormalities like elongated styloid processes have all been linked to an increased incidence of CAD [7].

Eagle’s syndrome is a rare cause of CAD, first described in 1937. It is characterised by the elongation of the styloid process or calcification of the stylomandibular or stylohyoid ligaments. An extended styloid process occurs incidentally in around 4% of the general population, but only approximately 4% of these individuals present with symptoms related to styloid elongation. Thus, the true incidence is approximately 0.16%, with a female-to-male ratio of 3:1. Patients are often older than 30 years and changes in the styloid process are usually bilateral. Depending on the angulation of the elongated styloid process, two clinical forms are described: typical stylohyoid syndrome and stylo-carotid artery syndrome. Symptoms of the stylo-carotid variety are caused by compression of the sympathetic chain and carotid artery. Syncope, ocular symptoms, facial pain, and cerebrovascular symptoms are all possible presentations. This variant's infrequent consequences include CAD.

Eagle syndrome is diagnosed based on a thorough medical history, physical examination, and elimination of other possible causes [3]. X-rays can diagnose Eagle’s syndrome but CT with three-dimensional reconstruction remains the gold standard. To diagnose CAD, a high index of clinical suspicion is required, and confirmation is achieved using Doppler ultrasonography, magnetic resonance imaging/magnetic resonance angiography, CT angiography, or catheter angiography. In most setups, CT angiography is readily available, easy to access, and acquires images quickly. It also has the added advantage of diagnosing both CAD and Eagle's syndrome. Medical therapy is frequently considered the first line of management in CAD secondary to Eagle’s syndrome. Aspirin, dipyridamole, or clopidogrel are used alone or in combination but there is no consensus regarding the superiority of one over the others. Several authorities believe that anticoagulation with heparin followed by warfarin therapy could be an acceptable option. Analgesics, anti-inflammatory agents, local steroids, and anaesthetic injections could be used for conservative management. Trans-pharyngeal manipulation and intraoral or trans-pharyngeal styloidectomy are alternative treatment options. Surgical intervention is not always feasible due to the risk of side effects or the condition of the patient [7-10].

Malignant middle cerebral artery infarction refers to space-occupying cerebral oedema after a middle cerebral artery infarct. Headache, nausea, and progressive focal deficits could be potential symptoms of malignant middle cerebral artery infarct. Treatment options include medications to reduce cerebral oedema and surgical decompression to relieve pressure effects. When this develops, the prognosis is typically poor, and trans-tentorial herniation and brainstem compression are frequently the causes of death. Unfortunately, our patient too developed a malignant middle cerebral artery infarct but recovered well after craniectomy, although he required extensive rehabilitation [11,12].

## Conclusions

Eagle's syndrome is a rare cause of stroke that many physicians are not familiar with, although it can be easily diagnosed with CT. Stroke in young patients or with unclear aetiology should prompt clinicians to consider atypical causes like Eagle's syndrome. Although Eagle’s syndrome is an uncommon cause of ischemic stroke, maintaining a high index of suspicion can be helpful in reaching the correct diagnosis. 
